# The autism/neuroprotection-linked ADNP/NAP regulate the excitatory glutamatergic synapse

**DOI:** 10.1038/s41398-018-0357-6

**Published:** 2019-01-15

**Authors:** Shlomo Sragovich, Anna Malishkevich, Yael Piontkewitz, Eliezer Giladi, Olga Touloumi, Roza Lagoudaki, Nikolaos Grigoriadis, Illana Gozes

**Affiliations:** 1 Lily and Avraham Gildor Chair for the Investigation of Growth Factors, Elton Laboratory for Neuroendocrinology, Department of Human Molecular Genetics and Biochemistry, Sackler Faculty of Medicine, Sagol School of Neuroscience and Adams Super Center for Brain Studies, Tel Aviv University, Tel Aviv, Israel; 2 The Alfredo Federico Strauss Center for Computational Neuroimaging, Tel Aviv University, Tel Aviv, Israel; 3 Department of Neurology, Laboratory of Experimental Neurology, AHEPA University Hospital, Aristotle University of Thessaloniki, Thessaloniki, Greece

## Abstract

Activity-dependent neuroprotective protein (ADNP), essential for brain formation, was discovered as a leading de novo mutated gene causing the autism-like *ADNP* syndrome. This syndrome is phenotypically characterized by global developmental delays, intellectual disabilities, speech impediments, and motor dysfunctions. The *Adnp* haploinsufficient mouse mimics the human *ADNP* syndrome in terms of synapse density and gene expression patterns, as well as in developmental, motor, and cognitive abilities. Peripheral ADNP was also discovered as a biomarker for Alzheimer’s disease and schizophrenia, with nasal administration of the ADNP snippet peptide NAP (enhancing endogenous ADNP activity) leading to partial cognitive and functional protection at the cellular, animal and clinical settings. Here, a novel formulation for effective delivery of NAP is provided with superior brain penetration capabilities. Also provided are methods for treating pertinent clinical implications such as autism, cognitive impairments, olfactory deficits, and muscle strength using the formulation in the *Adnp* haploinsufficient mouse. Results showed a dramatically specific increase in brain/body bioavailability with the new formulation, without breaching the blood brain barrier. Additional findings included improvements using daily intranasal treatments with NAP, at the behavioral and brain structural levels, diffusion tensor imaging (DTI), translatable to clinical practice. Significant effects on hippocampal and cerebral cortical expression of the presynaptic *Slc17a7* gene encoding vesicular excitatory glutamate transporter 1 (VGLUT1) were observed at the RNA and immunohistochemical levels, explaining the DTI results. These findings tie for the first time a reduction in presynaptic glutamatergic synapses with the autism/Alzheimer’s/schizophrenia-linked *ADNP* deficiency coupled with amelioration by NAP (CP201).

## Introduction

The neuroprotective drug candidate, NAP (NAPVSIPQ) also called davunetide (CP201) was derived from activity-dependent neuroprotective protein (ADNP) by structure activity screening^1^. We further identified the shared target of NAP and ADNP that is dependent on the SxIP = SIP domain, the signature motif binding microtubule end binding proteins EB1 and EB3^2^. NAP enhanced ADNP-EB3 interaction to promote dendritic spine formation and synaptic plasticity^2^, further augmenting Tau-microtubule interaction^3^. Additionally, NAP enhanced ADNP-microtubule-associated protein 1 light chain 3 (LC3) interaction^4^, thus protecting essential cellular/neuronal protective mechanisms such as axonal transport^5^, autophagy^6^, and inhibiting apoptosis^7^. In this respect, *Adnp* deficiency in mice resulted in impaired axonal transport which was ameliorated by the NAP active modified fragment SKIP^8^.

NAP showed neuroprotection in mouse models of chronic neurodegeneration such as Alzheimer’s disease^9–11^, Parkinson’s disease^12^, frontotemporal dementia^13^, amyotrophic lateral sclerosis (ALS)^5^, and diabetes-associated brain degeneration, suggestive as a risk factor for Alzheimer’s disease^14^. All of these pathologies are characterized by progressive neuronal cell death that is linked to deterioration of the microtubule system [e.g., Cash et al., 2003^15^].

Interestingly, the microtubule system is also deficient in psychiatric diseases, such as schizophrenia, with NAP showing protection against cognitive deficits in two microtubule-associated mouse models of schizophrenia namely, the microtubule-associated protein 6 deficient (*Map6*^+/−^) mouse^6^, and the mutated disrupted in schizophrenia 1 (DISC1) mouse^16^. Importantly, NAP also protected against acute brain injury (at the time of injury) in mouse models of head trauma^17^, stroke^18^, epilepsy^19^, and fetal alcohol syndrome [e.g., Spong *et al*., 2001^20^], providing long-lasting effects [e.g., Zaltzman et al., 2003^21^].

Together, these studies attest to preclinical efficacy, holding a promise for clinical effectiveness. Indeed, in clinical studies, NAP (davunetide) showed efficacy in two independent studies, namely, increase in cognitive scores in amnestic mild cognitive impairment patients^22^, and protection of functional activities of daily living in schizophrenia patients^23^ coupled to brain neuroprotection^24^. Nevertheless, in a study performed in severely affected individuals suffering from progressive supranuclear palsy (PSP), belonging to the family of rapidly progressing frontotemporal neurodegenerations, NAP (davunetide) did not show efficacy^25^, implicating potential insufficient target engagement.

For chronic non-invasive nasal NAP administration, we routinely used (7.5 mg NaCl, 1.7 mg citric acid monohydrate, 3 mg disodium phosphate dihydrate, 50% 1 mg benzalkonium chloride in 1 ml solution, termed DD)^26^, which yields significant brain bioavailability^27^. The benzalkonium chloride in the DD solution is a preservative needed for efficacious peptide drug delivery. While chlorobutanol has been in use as a preservative in various pharmaceutical formulations including nasal sprays, it was neither known nor understood that it had any notable effect on the absorption of a bioactive peptide drug, like NAP (CP201). Therefore, in the current study we simplified the excipient and exchanged the benzalkonium chloride with chlorobutanol (0.25% chlorobutanol, 0.85% NaCl, pH = 3.5 to 4.0, termed CB), revealing a significant ~4-fold increase in brain bioavailability, and a dramatic concentration in brain vs. body for NAP in chlorobutanol, thus showing a better penetration of NAP in its presence. It should also be made clear that for the most part (except for the comparison of bioavailability above), the current study is not a direct comparison between the two different vehicle formulations of DD vs. CB, but rather a verification of the profound effects of NAP administered with the new CB formulation.

To assess if NAP in chlorobutanol also provided neuroprotection, the new formulation was tested in the *Adnp* haploinsufficient mouse model depicting brain damage and behavioral deficits. While complete *Adnp* deficiency in mice is lethal and the brain does not form^28^, the *Adnp*^+/−^ mouse is viable and suffers brain damage and cognitive deficiencies, in a sex-dependent manner^8,29,30^.

In the human population, de novo mutations were recently identified in *ADNP*, in children with autism spectrum disorder with cognitive disabilities^30–32^. In the adult and aging population, *ADNP* and the sister *ADNP2* transcripts are deregulated in the postmortem schizophrenia hippocampus^33^. In lymphocytes, *ADNP* and *ADNP2* transcript levels can serve as biomarkers for schizophrenia^4^ and Alzheimer’s disease^34^. ADNP levels in the plasma are significantly correlated with IQ^34^. ADNP single polynucleotide polymorphisms (SNPs) have been associated with bipolar disorder with comorbid eating disorder [e.g., rs6096154 (C/T); rs6020824 (C/T); rs1062651 (A/G)]^35^.

In terms of genes regulated by/associated to ADNP, [1] de novo mutations in the ADNP-binding CBX5 (HP1-alpha^36^) have been linked to schizophrenia^37^. [2] ADNP regulates calcium channel (CACNA1C) expression in a sex-dependent manner^8^. In schizophrenia-spectrum affected males, rs10774035 minor allele (T) carriers had higher Global Assessment of Functioning (GAF) scores at three time points (premorbid, worst ever, current). In contrast, females carrying rs10774035 minor alleles had impaired recovery from schizophrenia-spectrum episodes^38^. [3] Furthermore, *ADNP* regulates the expression of apolipoprotein E (*APOE*), the major risk gene for Alzheimer’s disease, in a sex-dependent manner^30^.

Together, this involvement of ADNP in autism, schizophrenia, and Alzheimer’s disease, makes the *Adnp* haploinsufficient mouse (*Adnp*^+/−^)^8^ an interesting model for further studies of drug efficacy. Here, NAP-chlorobutanol treatment provided highly significant protection. Given the broad association of ADNP with human brain disease, we foresee a wide range of clinical applications for the new NAP-davunetide (CP201)—chlorobutanol formulation, as well as additional pipeline products^8^.

## Materials and methods

### NAP formulation

NAP (NAPVSIPQ) was synthesized as previously described [e.g., Vaisburd et al., 2015^16^]. For bioavailability studies, NAP was labeled with cy 5.0 on the amino terminal site in the lab of Prof. Doron Shabat from the school of chemistry (Tel Aviv University). The labeled peptide was dissolved in 1XDD/ml solution (7.5 mg NaCl, 1.7 mg citric acid monohydrate, 3 mg disodium phosphate dihyrate, 50% 1 mg benzalkonium chloride in 1 ml solution) or in chlorobutanol solution containing 0.25% chlorobutanol, 0.85% NaCl, pH = 3.5 to 4.0. Chlorobutanol (designated below as CB, http://www.athenstaedt.de/englisch/chemikalien/haupt.htm) is a well-accepted, widely used, very effective preservative in many pharmaceuticals and cosmetic products, e.g., injections, ointments, products for eyes, ears and nose, and dental preparations, etc. It has antibacterial and antifungal properties and has been used for more than 125 years since it was first manufactured. Chlorobutanol is typically used at a concentration of 0.5% where it lends long-term stability to multi-ingredient formulations. Chemical formula: Hemihydrate: C4H7CI3O •1/2 H2O, Anhydrous: C4H7CI3O. Formula Weight: Hemihydrate: 186.47, Anhydrous: 177.46. The preferred formulation described here is comprised of 0.25% weight/weight of chlorobutanol, 0.85% sodium chloride, and 98.86% purified water, and the pH is ~3.5–4.0 http://www.faqs.org/patents/app/20110009321^39^.

### Animals

The *Adnp*^+/−^ mice, on a mixed C57BL and 129/Sv background, were previously described^28–30^. For continuous breeding, an ICR outbred mouse line was used^8,30^. Animals were housed in a 12-h light/12-h dark cycle animal facility, with free access to rodent chow and water. Genotyping was performed by Transnetyx (Memphis, TN, USA). Animal group sizes were determined in a pilot study, and animals were randomly allocated into experimental groups before the experiment. Blinded experienced researchers performed independently the different methodologies described in the manuscript, and repeated these successfully, thus substantiating the results. The animals were administered with different formulations, and further analyzed by different techniques involving brain bioavailability assessment (female ICR mice: DD-treated group *N* = 3; CB-treated group *N* = 3), blood brain barrier intactness (males: *Adnp*^+/+^ *N* = 3; *Adnp*^+/−^ *N* = 3, females: *Adnp*^+/+^ *N* = 3; *Adnp*^+/−^ *N* = 3), motor (males: *Adnp*^+/+^ *N* = 4; *Adnp*^+/−^ *N* = 3; *Adnp*^+/−^ NAP, *N* = 4, females: *Adnp*^+/+^ *N* = 3; *Adnp*^+/−^ *N* = 4; *Adnp*^+/−^ NAP, *N* = 4) and cognitive tests (males: *Adnp*^+/+^ *N* = 9–12; *Adnp*^+/−^ *N* = 7–13; *Adnp*^+/−^ NAP, *N* = 7–14, females: *Adnp*^+/+^ *N* = 3–4; *Adnp*^+/−^ *N* = 4; *Adnp*^+/−^ NAP, *N* = 3–4), diffusion tensor imaging (DTI; males: *Adnp*^+/+^ *N* = 6; *Adnp*^+/+^ NAP *N* = 6; *Adnp*^+/−^ *N* = 4; *Adnp*^+/−^ NAP, *N* = 4), as detailed below. At the end of the in vivo study, a subset of these mice was sacrificed and hippocampal/cortical RNA was extracted and subjected to quantitative RT-PCR (males: *Adnp*^+/+^ *N* = 4; *Adnp*^+/−^ *N* = 4; *Adnp*^+/−^ NAP, *N* = 4, females: *Adnp*^+/+^ *N* = 4; *Adnp*^+/−^ *N* = 4; *Adnp*^+/−^ NAP, *N* = 3), whereas a second subset of mice was sacrificed and further subjected to immunohistochemistry (males: *Adnp*^+/+^ *N* = 5; *Adnp*^+/−^ *N* = 5; *Adnp*^+/−^ NAP, *N* = 5). Biological replicates were used for all the in vivo procedures described in the manuscript, as well as for gene expression analysis, whereas technical replicates were used for immunohistochemistry. Outlier values were determined and excluded by Grubbs’ test (as described below in the “Statistical Analysis” section).

### In vivo imaging assessing immediate brain bioavailability

Eight-week-old female ICR mice were anesthetized by intraperitoneal injection of 10% Ketamine/5% xylazine in saline (0.1 ml/10gr). Sedation maintenance after 60 min was performed by subcutaneous injection of 20% Ketamine (0.5 ml/10gr). Following intranasal application of 0.1 mg/6ul, (DD or chlorobutanol vehicles), the mice were placed in the Maestro machine (Cri MaestroTM in vivo imaging system, a product of Cambridge Research & Instrumentation, Inc. CRi 35-B, Woburn, MA, USA). Light emission was measured every 15 min. Measures were taken for 2 h, after which, the animals were sacrificed, brains were removed, and placed in the Maestro for measurements of light emission (excitation = 670 nm, emission = 700 nm).

### Blood brain barrier intactness

Three–five-month-old male and female ICR or *Adnp*^+/+^/*Adnp*^+/−^ mice on an ICR background^40^ were pre-treated with intranasal chlorobutanol formulation (5 µl/nostril) or saline for control animals. Two hours after nasal administration, Evans blue detection (a marker for blood brain barrier intactness) was performed by intraperitoneal injection of 2% of the dye (4 ml/kg, 120 µl/30 gr mouse). Two hours after Evans blue injection, the animals were sacrificed. The animals were perfused with ice-cold saline through the left ventricle for 20 min to remove residual intravascular agent. The brains were removed for further dissection and assays. Quantitative evaluation of the Evans blue dye was performed using a previously published method^41^. Briefly, each tissue sample was weighed, homogenized in a three-fold volume of 50% trichloroacetic acid (wt/volume) solution (6.1 N solution; Sigma), and centrifuged at 10,000 r.p.m. for 20 min. The supernatants were diluted with ethanol (1:3), and fluorescence was quantified by using a microplate fluorescence reader (Victor2-V multilabel plate reader, PerkinElmer, Wellesley, MA, USA), (excitation: 620 nm, emission: 680 nm). Sample value calculations were based on Evans blue dye standards mixed with the same solvent (0–50 ng/ml). Results were expressed in nanograms of Evans blue dye per milligram of tissue.

### NAP behavioral measurements in the *Adnp*^+/−^ mice

Experiments were performed as previously described^8,30,40^. Three–six-month-old male mice were used, and NAP-CB was administered once daily (0.5 µg/5 µl/mouse) for one month. The behavioral assays were then initiated together with a continuous drug application. For detailed description of the object recognition test and the social approach task, please see supplemental materials and methods. In the odor discrimination test, odors were presented on a suspended cotton swab to the test mouse placed into the clean cage with fresh shavings. Each mouse was tested during three consecutive 2-min periods for each odor, with 2-min intervals between presentations. The time that the mouse smelled the swab was recorded (beginning whenever the animal oriented its nostrils toward the cotton swab, within 2 cm or less)^8,30^. The hanging wire test measuring the strength of the mouse paws by using the latency to fall off an inverted cage lid (placed 50 cm above the surface) onto a soft bedding (maximum time 90 sec), was performed as previously described^5,40^. Data are expressed as mean ± SEM.

### Magnetic resonance imaging (MRI) assessing chronically treated brains

Seven–eight-month-old male mice underwent MRI on a 7.0 T/30 spectrometer (Bruker, Rheinstetten, Germany) using a volume coil for excitation and a rat quadrature coil for acquisition. The MRI protocol used here was the DTI.

DTI was obtained using a diffusion-weighted (DW) spin-echo echo-planar-imaging (EPI) pulse sequence with the following parameters: TR/TE = 4000/25 ms, *Δ/δ* = 10/4.5 ms, 4 EPI segments and 32 non-collinear gradient directions with a single *b* value (1000 sec/mm^2^) and two images with b value of 0 sec/mm^2^ (referred to as b0). Geometrical parameters were: 24 slices of 0.5 mm thickness, matrix size of 128 × 128 and FOV of 20 mm^2^). The imaging protocol was repeated three times for signal averaging and to compensate for acquisition where significant head motion was observed. Image analysis included DTI analysis of the DW-EPI images to produce the mean diffusivity (MD) and fractional anisotropy (FA) indexed maps following a two-way ANOVA with Tukey post-hoc test.

### Gene expression analysis

Hippocampal and cerebral cortical RNA of mouse that were 7.5-month-old were extracted using TRI Reagent® (T9424, Sigma-Aldrich, MO, USA). A volume of 1 μg RNA/sample was then subjected to reverse transcription (RT) using qScript cDNA Synthesis Kit (Quanta Biosciences, Gaithersburg, MD, USA). Further Real-time PCR analysis was performed using PerfeCta^TM^ SYBR® Green FastMix^TM^, Low ROX^TM^ (Cat. No. 95074–012, Quanta Biosciences, Gaithersburg, MD, USA) and the QuantStudio 12 K Flex Real-Time PCR System (Thermo Fisher Scientific, Waltham, MA, USA). RNA expression levels were determined using specific mouse primers: *Slc17a7* gene encoding VGLUT1, sense 5′-CTATGTCTATGGCAGCTTCG-3′, anti-sense 5′-TCAATGTATTTGCGCTCCT-3′. Hypoxanthine-guanine phosphoribosyltransferase (*Hprt*) was selected as a stable reference gene with appropriate primers for mouse, sense 5′-GGATTTGAATCACGTTTGTGTC-3′, anti-sense 5′-AACTTGCGCTCATCTTAGGC-3′. Results are presented as 2^-ΔCT^^42^.

### Immunohistochemistry

Immunohistochemistry was carried out as previously described^8^. Briefly, paraffin sections were deparaffinized, rehydrated in graded alcohols, and antigen retrieval was performed with citrate buffer pH 6. Endogenous peroxidase was blocked with 3% H_2_O_2_ in methanol and was followed by incubation in the appropriate blocking buffer for 30 min. Sections were incubated overnight at 4 °C with the primary antibodies against VGLUT1 (Santa Cruz Biotechnology, sc-13320). The secondary antibody used was Rabbit Anti-Goat IgG (EMD Millipore, AP106B). Immunoreactions were visualized with the aim of Avidin-Peroxidase (Sigma- Aldrich Chemical), and 3,3′-Diaminobenzidine (DAB, brown color, Sigma-Aldrich Chemical) was utilized as chromogen. Sections were then counterstained with hematoxylin (light blue color).

Sections were further examined under optical microscope Zeiss Axioplan-2 with the aid of a CCD camera (Nikon DS-5M) for observations of the slides. In average, 25 optical fields were examined from each group under a magnification of ×40. Measurements were performed with Image J software (1.43 u) in the areas of the hippocampus and the cortex. The results were expressed as area/mm^2^ (as previously described^14^), representing the area of DAB positive signal in each photo divided by the total area of the same photo. An additional parameter measured was the Integrated Density, calculating and displaying two values: the product of Area and Mean Gray Value.

### Statistical analysis

Results are presented as means ± standard error of the mean (SEM). Data were checked for normal distribution by normality test. For two different categorical independent variables, two-way analysis of variance (ANOVA) or two-way repeated measures ANOVA followed by the Tukey post-hoc test were performed. Unpaired Student’s *t*-test or Mann-Whitney *U* test analyses were performed when needed. All determinations were made with a 95% confidence interval, and *P*-values smaller than 0.05 were considered significant. All tests were two-tailed. For in vivo procedures, gene expression analysis, and immunohistochemistry, outlier values were excluded using the Graphpad outlier calculator (https://graphpad.com/quickcalcs/Grubbs1.cfm). All statistical analyses were conducted using SigmaPlot software version 11 Inc. for Windows (Chicago, IL, USA). For immunohistochemistry, data analysis was performed using the Graph Pad Prism 7.0 software. The normality was tested using the Shapiro–Wilk and Kolmogorov–Smirnov tests. Parametric data were analyzed using one-way ANOVA with Holm-Sidak’s multiple comparisons test. Non-parametric data were analyzed using the equivalent Kruskall–Wallis test followed by Dunn’s multiple comparison test.

### Study approval

All procedures involving animals were conducted under the supervision and approval of the Animal Care and Ethics Committee of Tel Aviv University and the Israeli Ministry of Health (M-15–059).

## Results

### Chlorobutanol (CB) dramatically enhances brain penetration

**Fig. 1:**
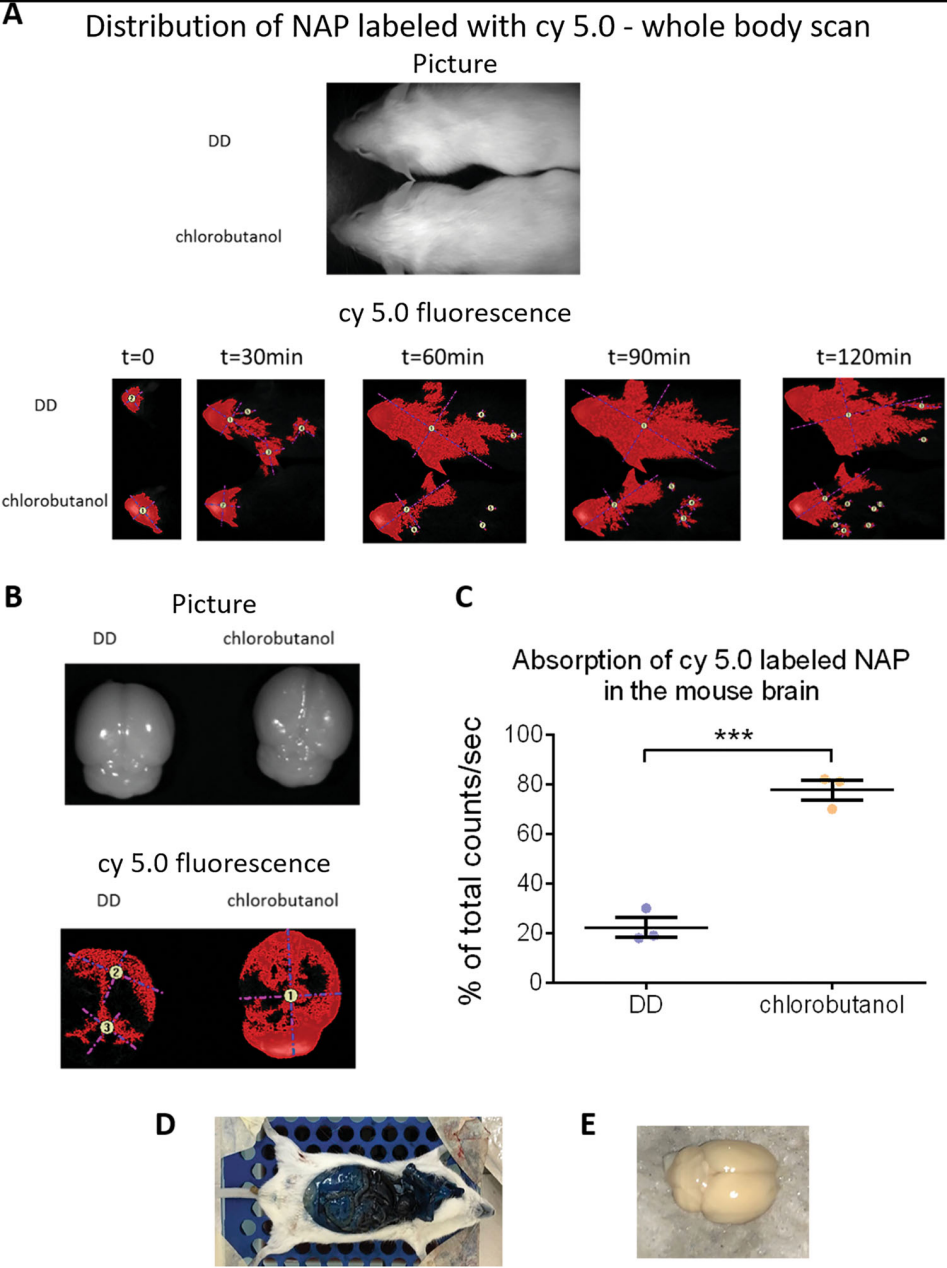
The presence of chlorobutanol in the formulation results in increased NAP concentration in the brain. Experiments were performed as described in the Materials and Methods section (*n* = 3 per experimental group). **a** The upper panel shows the picture of two mice and the lower panel shows the Maestro results after the application of labeled NAP. **b** The upper panel shows the picture of two mouse brains and the lower panel shows the Maestro results, after the application of labeled NAP, decapitation and brain removal. **c** The presence of chlorobutanol in the formulation results in 4-fold more NAP in the brain. The Maestro results are shown after the application of labeled NAP, decapitation and brain removal. **d** Representative picture showing substantial Evans blue dye in peripheral organs. **e** Representative picture showing the intact brain of the animal presented in (**d**), showing no Evans blue dye penetration

Figure 1a shows enhanced brain/body bioavailability in the presence of the newly tested vehicle, chlorobutanol over time. Fig. 1b displays enhanced brain bioavailability by picture evaluation. Fig. 1c shows quantitative assessment of three independent experiments, revealing the dramatic 4-fold increase in specific brain bioavailability. Further results showed no significant brain penetration of Evans blue ( < 0.1 ng/ml, which was the limit of detection), and no difference between chlorobutanol and saline. In an additional set of experiments, neither genotype nor sex differences were observed. Representative pictures show penetration of the Evans blue dye in the periphery (Fig. 1d), but not in the brain (Fig. 1e), with brain concentration values < 0.03 ng/ml (Supplemental Fig. S1). These findings provide clear evidence that the blood brain barrier remained intact in the presence of chlorobutanol.

### NAP-CB treatment increases the relative discrimination between novel and familiar objects and further increases social memory

**Fig. 2:**
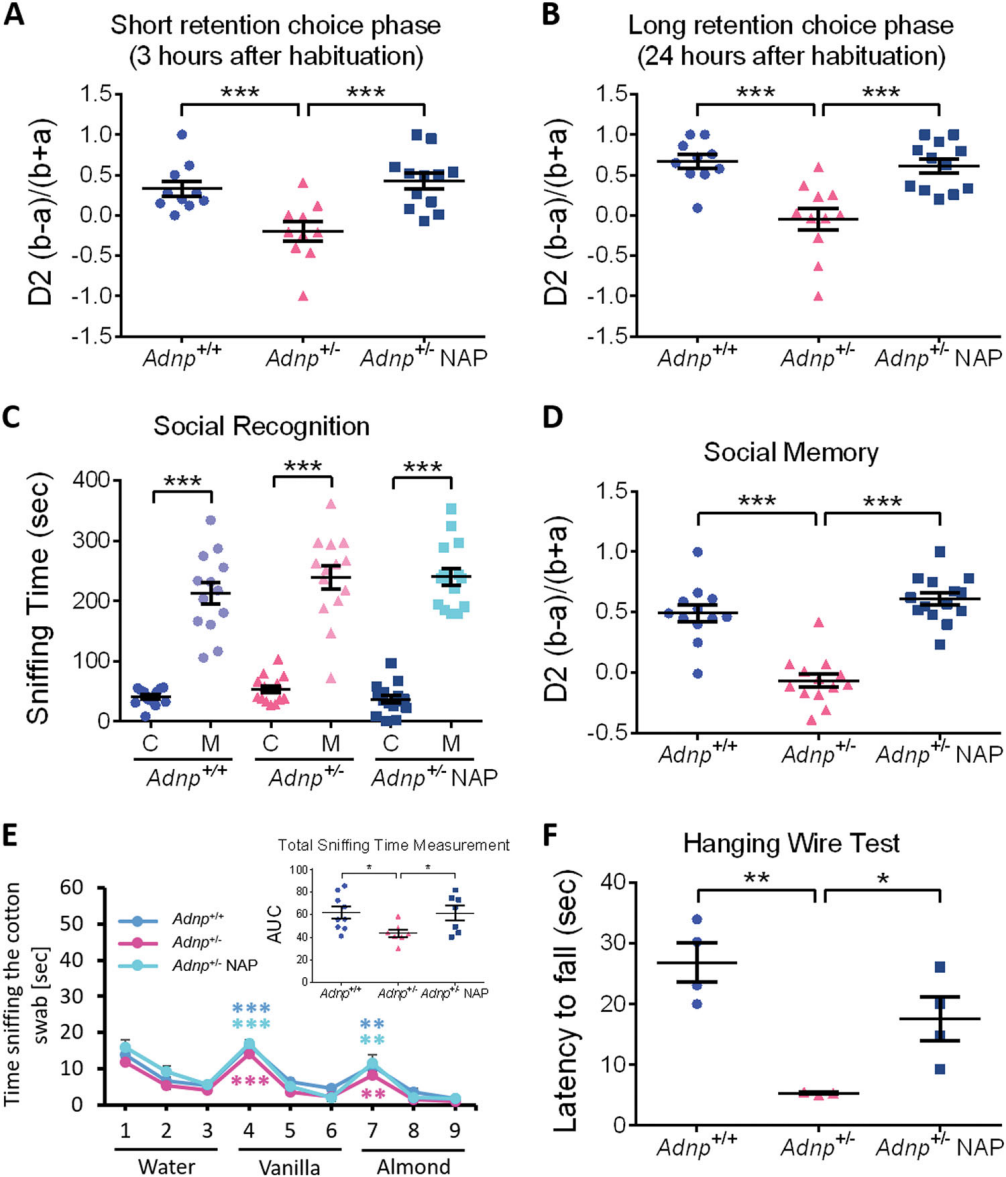
NAP treatment increases the relative discrimination between novel and familiar objects, protects social memory and preserves motor function. For males, animal performance in the behavioral tests is shown (*n* = 3–15 per experimental group). Data are expressed as mean ( ± SEM) total time (sec) spent exploring all objects/mice designated by relative discrimination index (D2, ‘a’ and ‘b’ - exploration of familiar and novel objects/mice, respectively). **a**, **b** For both short and long retention choice phases in males, Two-way ANOVA with Tukey post-hoc test was performed. For short retention choice phase, main genotype (F(1,38) = 4.702, *p* = 0.036), treatment (F(1,38) = 9.478, *p* = 0.004) and interaction (F(1,38) = 9.732, *p* = 0.003) effects were found, with significant differences between *Adnp*^+/+^ and *Adnp*^+/−^ mice (***p < 0.001), and between *Adnp*^+/−^ and NAP-treated *Adnp*^+/−^ mice (***p < 0.001). For long retention choice phase, main genotype (F(1,38) = 9.327, *p* = 0.004), treatment (F(1,38) = 6.236, *p* = 0.017) and interaction (F(1,38) = 15.279, *p* < 0.001) effects were found, with significant differences between *Adnp*^+/+^ and *Adnp*^+/−^ mice (***p < 0.001), and between *Adnp*^+/−^ and NAP-treated *Adnp*^+/−^ mice (***p < 0.001). **c** In the social recognition test, main effect for sniffed item was found (F(1,39) = 302.179, *p* < 0.001), with significant differences between sniffing time of the cup (**c**) and mouse (M) in *Adnp*^+/+^, *Adnp*^+/−^, and NAP-treated *Adnp*^+/−^ mice (**p < 0.01), and NAP-treated *Adnp*^+/−^ mice (***p < 0.001 vs. cup). Two-way repeated measures ANOVA with group as a fixed factor and sniffed item (e.g., mouse vs. cup) as repeated factor and Tukey post-hoc test was performed. **d** *Adnp*^+/−^ male mice spent less time in exploring the novel mouse, as compared with *Adnp*^+/+^ mice. Treatment with NAP improved social memory for the *Adnp*^+/−^ mice. Unpaired Student’s *t*-test analyses revealed significant differences between vehicle-treated *Adnp*^+/+^ and *Adnp*^+/−^ mice, and between NAP- and vehicle-treated *Adnp*^+/−^ mice (***p < 0.001). All reported p-values were also significant after multiple comparisons correction at FDR of 10%. **e** In males, the olfactory function was preserved, and no significant differences were observed between experimental groups. **p < 0.01, ***p < 0.001 vs. previous sniffing (novel vs. familiar odor), paired *t*-test. For each experimental group, general olfaction ability was measured by calculating area under the curve (AUC)—inset graph. Unpaired Student’s *t*-test analyses revealed significant differences between vehicle-treated *Adnp*^+/+^ and *Adnp*^+/−^ mice, and between NAP- and vehicle-treated *Adnp*^+/−^ mice (*p < 0.05). **f** In the Hanging Wire Test, male *Adnp*^+/−^ mice display significant decreased latency to fall, with NAP ameliorating. Unpaired Student’s *t*-test analyses revealed significant differences between vehicle-treated *Adnp*^+/+^ and *Adnp*^+/−^ mice, and between NAP- and vehicle-treated *Adnp*^+/−^ mice (**p < 0.01, *p < 0.05). All reported *p*-values were also significant after multiple comparisons correction at FDR of 10%

Animal performance in the object recognition memory test is shown. Two identical objects were presented during the habituation phase, with one of the identical objects replaced by a novel object during the short retention choice phase (3 h), and the long retention choice phase (24 h). *Adnp*^+/−^ mice compared with *Adnp*^+/+^ mice spent significantly shorter time periods in exploring the new objects, indicative of impaired memory, with intranasal NAP-CB treatment completely ameliorating this impairment (Fig. 2a, b).

While no effect was observed in the *Adnp*^+/−^ male mouse social recognition, with preference to mice rather than objects (Fig. 2c), *Adnp* haploinsufficiency showed significantly inhibited social memory, which was completely ameliorated by NAP-CB treatment (Fig. 2d). We have previously shown essentially no significant NAP effects on behavior in the *Adnp*^+/+^ mice^40^. This was repeated here specifically in the object memory test (Supplemental Fig. S2A, B). We have also extended the experiments to female mice, and interestingly, in the social recognition test CB- or NAP-treated females displayed significant preference to mice rather than objects (Supplemental Fig. S2C), unlike previous findings with the “DD” formulation^8,30^. Nevertheless, as previously observed^30,40^, *Adnp*^+/+^ females tended to be less interested in the novel mouse (Supplemental Fig. S2D), compared with *Adnp*^+/+^ males (Fig. 2d). Furthermore, in both sexes, *Adnp* haploinsufficiency showed significantly inhibited social memory, which was completely ameliorated by NAP treatment (Fig. 2d, males and Supplemental Fig. S2D, females).

### *Adnp* deficiency affects olfaction, muscle strength, and gene expression: amelioration by NAP treatment

As social behavior depends on olfaction, this was also measured showing intact odor discrimination ability in CB-treated males (Fig. 2e) and a sex difference, with no preference for a specific olfactory cue in CB-treated females (Supplemental Fig. S2E), thus corroborating previous findings observed for DD-treated mice^8,30^. Interestingly, in CB-treated males, *Adnp* haploinsufficiency showed a reduction in the total time spent with the different odors, which was significantly increased upon NAP treatment (Fig. 2e, inset). As opposed to this, in females, CB-treated *Adnp* haploinsufficient mice showed significant increased total odor sniffing time, which was significantly reduced by NAP treatment (Supplemental Fig. S2E, inset).

Given the fact that children carrying *ADNP* mutations (*ADNP* syndrome children) exhibit motor impairments^31^, we also utilized the hanging wire test to measure potential impairments and amelioration by NAP. Results showed a significant impairment due to *Adnp* haploinsufficiency and amelioration by NAP treatment (Fig. 2f). Interestingly, a sex difference was observed here as well, with females showing better performance compared with males (Supplemental Fig. S2F) and as previously observed^40^.

### Structural changes in the *Adnp*^+/−^ brains and amelioration by NAP treatment

**Fig. 3:**
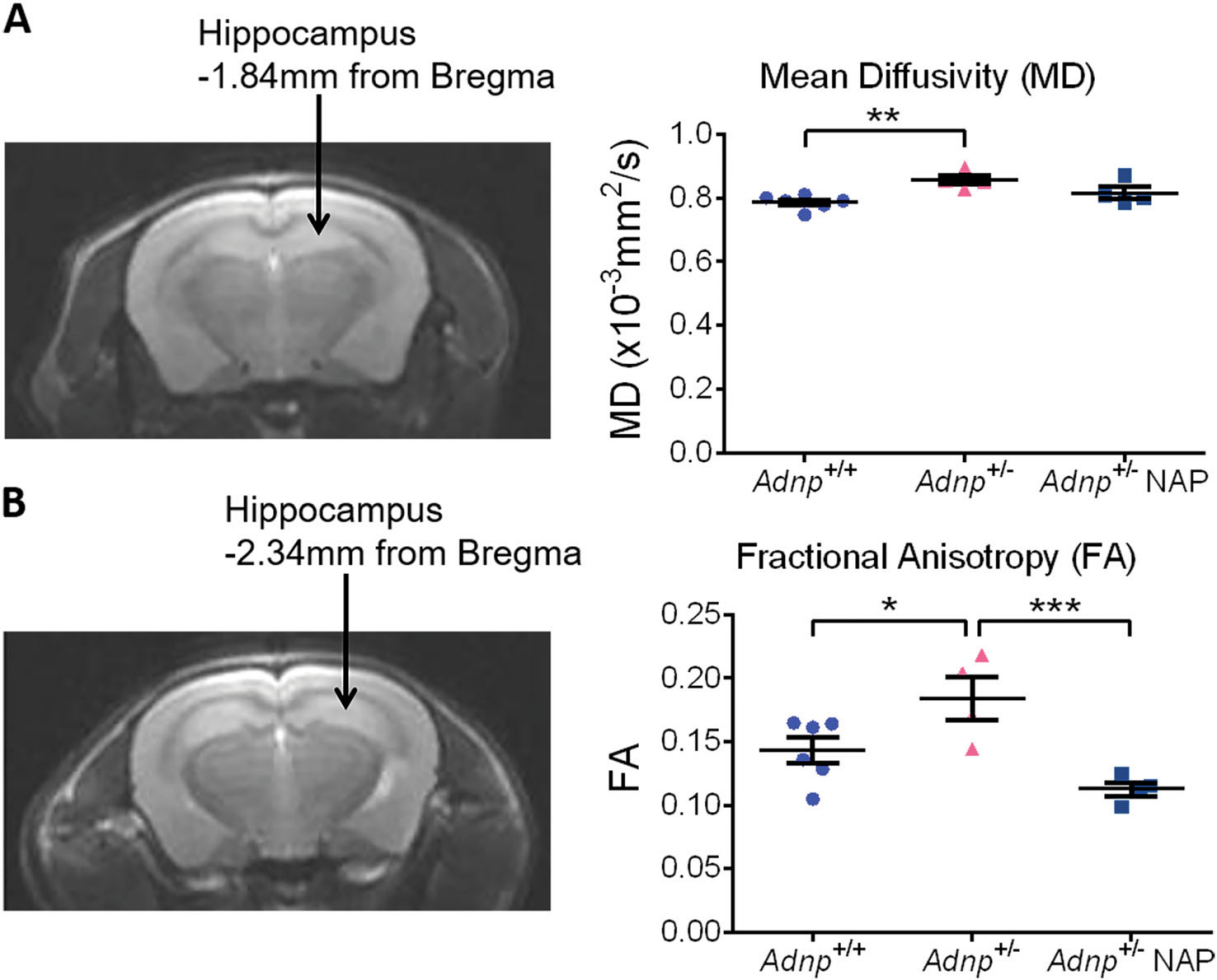
NAP protects against increases in hippocampal MD and FA in the *Adnp*^+/−^ mice. Two-way ANOVA with Tukey post-hoc test was performed (*n* = 4–6 per experimental group). **a** For mean diffusivity (MD), a representative T2-weighed image at the level of the hippocampus at −1.84 mm from Bregma is presented. Main genotype (F(1,16) = 8.775, *p* = 0.009) and interaction (F(1,16) = 4.956, *p* = 0.041) effects were found, with a significant increased MD in chlorobutanol (CB)-treated *Adnp*^+/−^ mice, as compared with their *Adnp*^+/+^ control mice (**p < 0.01). This increase, although insignificant, was slightly reduced in NAP-treated *Adnp*^+/−^ mice. **b** For fractional anisotropy (FA), a representative T2-weighed image at the level of the hippocampus at −2.34 mm from Bregma is presented. Main treatment (F(1,16) = 12.782, *p* = 0.003) and interaction (F(1,16) = 9.986, *p* = 0.006) effects were found, with a significant increased FA in chlorobutanol (CB)-treated *Adnp*^+/−^ mice, as compared with their *Adnp*^+/+^ control mice (*p < 0.05). This increase was significantly reduced in NAP-treated *Adnp*^+/−^ mice (***p < 0.001)

Diffusion MRI, and specifically the DTI is regarded as a microstructural probe^43,44^. From the DTI data, two indices were extracted to study brain structures, namely the MD and FA. MD describes the rotationally invariant magnitude of water diffusion within brain tissue and is used to examine differences of brain structure^43,45,46^. MD is also sensitive to cellularity, edema, and necrosis, and differences in it could reflect variations within the intra- and extracellular space^47,48^, a reduction in neuropil^49^, and/or index global increases in cerebrospinal fluid (CSF)^50^. Increased MD is indicative of increased brain tissue damage^51,52^. Here, *Adnp* haploinsufficiency in male mice has led to a significant increased MD in the hippocampus (Fig. 3a). However, neither *Adnp*^+/−^ (Fig. 3a) nor *Adnp*^+/+^ mice (Supplemental Fig. S3A) were affected by NAP treatment. Furthermore, FA which is used to characterize the organization of white matter fibers^53^, was shown to be significantly increased in *Adnp*^+/−^ mice, thus implying of structural impairment. Importantly, this DTI observed structural impairment was ameliorated by NAP-CB treatment (Fig. 3b). FA measurements in *Adnp*^+/+^ mice treated with NAP were not affected (Supplemental Fig. S3B).

### Immunohistochemical changes in the *Adnp*^*+/−*^ brains correlate with reduced cognition and ameliorated by NAP treatment

Our original cell culture results showed NAP protection against excitotoxicity over a broad concentration range, suggesting an involvement of the glutamatergic system in ADNP/NAP activity^1^.

Furthermore, the glutamatergic system is known to be involved in brain shaping, e.g., glutamine triggering long-lasting increase in striatal network activity in vitro^54^, with longitudinal imaging revealing subhippocampal dynamics in glutamate levels associated with histopathologic events in mice^55^. Therefore, we asked whether the *Adnp* genotype impacts these key neurotransmission systems. Also, as our previous experiments, labeling dendritic spines in vivo, showed reduction in spine density in the hippocampus and the cortex as a consequence of *Adnp* deficiency and protection by NAP injection^40^, we now sought to determine possible genotype/treatment/sex effects on the vesicular glutamate transporter VGLUT1. VGLUT1 is both necessary and sufficient for uptake and storage of glutamate, and thus comprises the sole determinant for an excitatory glutamatergic phenotype^56^. VGLUT1 is further implicated in behavioral flexibility, impaired in mental diseases, plays a role in synaptic plasticity and excitotoxicity, as well as regulates presynaptic pH^57,58^.

In females, hippocampal VGLUT1 was not affected by the *Adnp* genotype or NAP treatment (Supplemental Fig. S4A). In the cerebral cortex, female *Adnp*^+/−^ mice exhibited significantly reduced VGLUT1 expression, with no effect for NAP (Supplemental Fig. S4B).

**Fig. 4:**
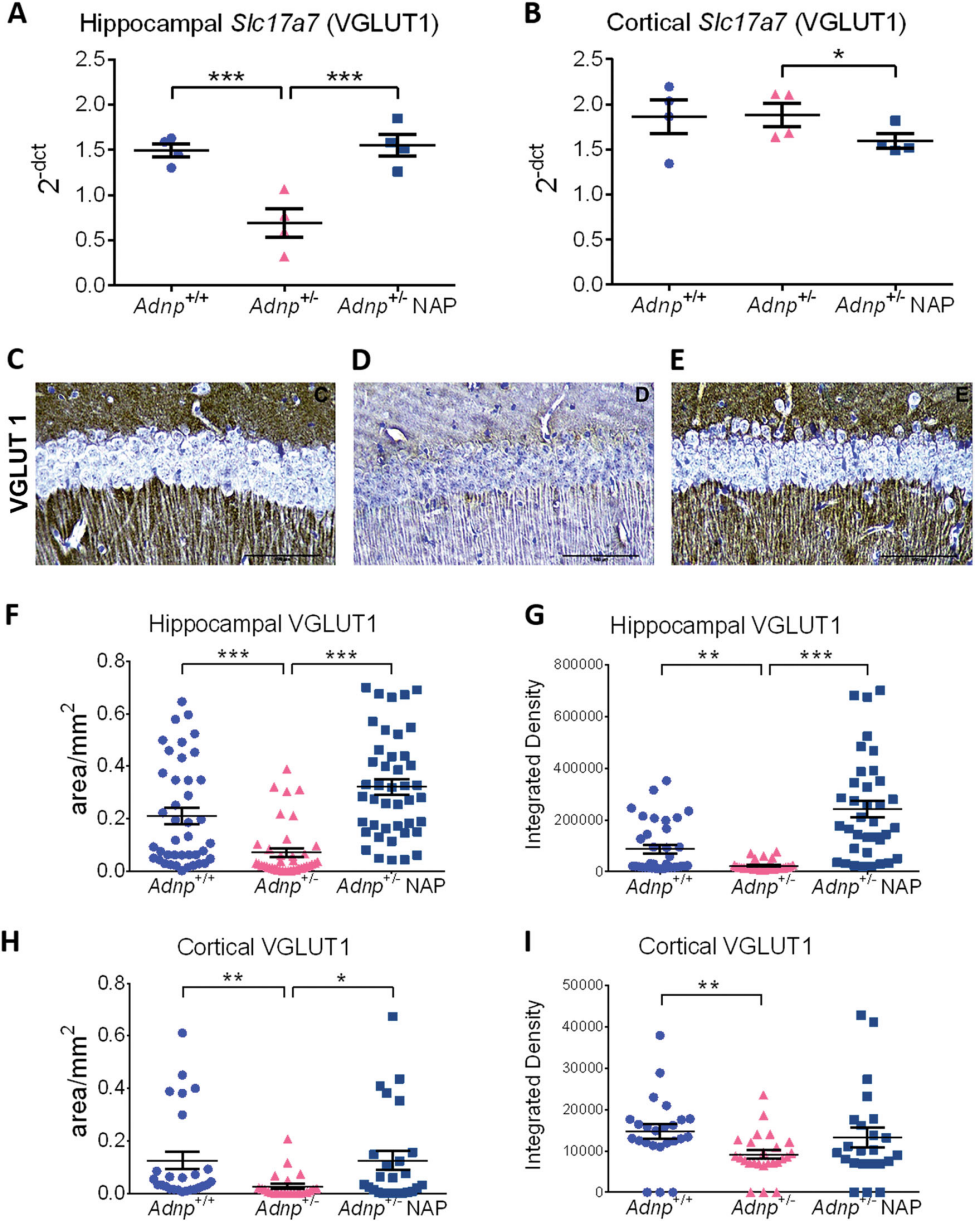
*Adnp* deficiency results in *Slc17a7* (VGLUT1) gene and protein expression alterations: NAP treatment significantly affects VGLUT1 expression in the hippocampus and cerebellar cortex. For gene expression analysis, results are presented as 2^−ΔCT^, normalized to *Hprt* (*n* = 4 per experimental group). VGLUT1 gene expression was significantly affected in the hippocampus (**a**) and cerebral cortex (**b**). Unpaired Student’s *t*-test analyses revealed significant differences between vehicle-treated *Adnp*^+/+^ and *Adnp*^+/−^ mice, and between NAP- and vehicle-treated *Adnp*^+/−^ mice (*p < 0.05, ***p < 0.001). All reported *p*-values were also significant after multiple comparisons correction at FDR of 10%. For immunohistochemistry, technical replicates obtained from five animals per group are presented (*n* = 24–43 replicates per experimental group). **c**–**e** Immunohistochemical representative pictures for the protein expression of VGLUT1 from the hippocampal area. The positive VGLUT1 signal is the brown color (DAB staining), whereas the light blue color is the nuclei cells, counterstained with hematoxylin, scale bar 100 μm. **f** The parameter of area/mm^2^, representing the area of DAB positive signal in each photo divided by the total area of the same photo, is presented in the graph. Differences among the three groups are shown, with significantly decreased hippocampal VGLUT1 expression in chlorobutanol (CB)-treated *Adnp*^+/−^ mice, compared with *Adnp*^+/+^ and NAP-treated *Adnp*^+/−^ mice (***p < 0.001). **g** The parameter of Integrated Density, calculating and displaying two values: the product of Area and Mean Gray Value, is presented in the graph. Differences among the three groups are shown, with significantly decreased hippocampal VGLUT1 integrated density in chlorobutanol (CB)-treated *Adnp*^+/−^ mice, compared with *Adnp*^+/+^ (**p < 0.01) and NAP-treated *Adnp*^+/−^ mice (***p < 0.001). **h** Significant differences in area counting were observed with cortical VGLUT1 expression, between CB-treated *Adnp*^+/+^ and *Adnp*^+/−^ mice (**p < 0.01), and CB- vs. NAP-treated *Adnp*^+/−^ mice (*p < 0.05). **i** Significant differences in integrated density were observed with cortical VGLUT1 expression, between CB-treated *Adnp*^+/+^ and *Adnp*^+/−^ mice (**p < 0.01). All reported *p*-values were also significant after multiple comparisons correction at FDR of 10%

In males, complementing DTI data (Fig. 3) our results (Fig. 4a) revealed that in *Adnp*^+/−^ mice, *Slc17a7* (VGLUT1*)* gene expression was significantly decreased in the hippocampus, and completely reversed by NAP treatment (Fig. 4a), while in the cerebral cortex, NAP treatment resulted in a small, albeit significant decrease in the VGLUT1 transcript (Fig. 4b). At the protein level, a significant reduction in VGLUT1 was observed in both the hippocampus (Fig. 4c–e, immunohistochemistry, 4F-G, densitometry) and cerebral cortex (Fig. 4h, i, densitometry). When using area counting, NAP treatment was shown to provide full protection against VGLUT1 decreases in both the hippocampus and the cerebral cortex (Fig. 4f, h), whereas in terms of intensity changes in VGLUT1 expression, NAP effect was significant in the hippocampus (Fig. 4g), and exhibited a trend of improvement in the cortex (Fig. 4i).

**Fig. 5:**
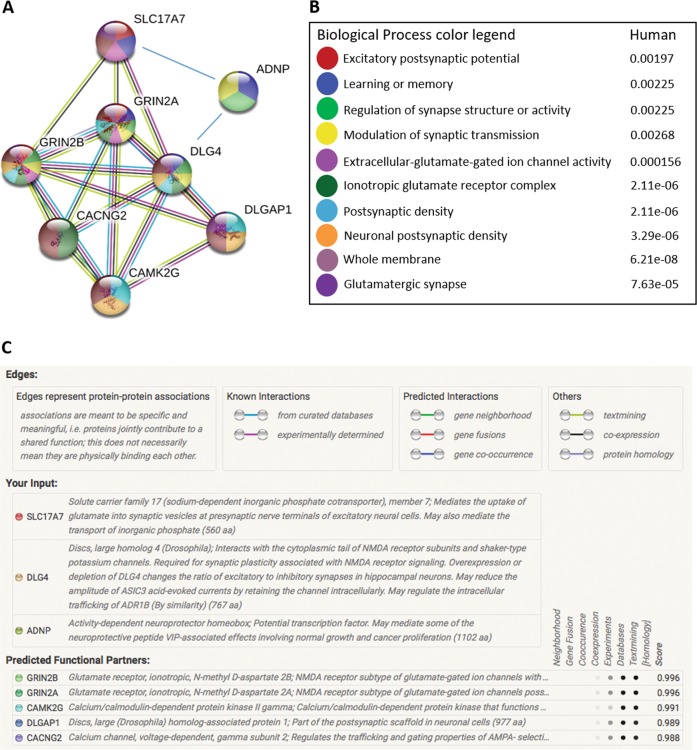
Function enrichment and network analysis indicate strong regulation of the glutamatergic synapse. STRING protein-protein interaction network^67^ (https://string-db.org) was performed for ADNP, DLG4, and SLC17A7 in the human database (blue lines) (**a**). Enriched biological processes are marked on the network according to the color legend (**b**). Edges represent protein-protein associations, meant to be specific and meaningful with associated proteins jointly contributing to a shared function. This does not necessarily mean that the proteins are physically binding each other (**c**)

Our current data connected for the first time VGLUT1 regulation to ADNP/NAP, specifically, in the male hippocampus. In this respect, our previous data associated postsynaptic density protein 95 (PSD95, also known as DLG4) with ADNP/NAP activity^2,40^. Looking at potential additional protein interactions, we resorted to STRING analysis (Fig. 5). This revealed additional interacting proteins including [1] the glutamate receptor, ionotropic, N-methyl D-aspartate 2 A and 2B; NMDA receptor (NMDAR) subtype of glutamate-gated ion channels with high calcium permeability and voltage-dependent sensitivity to magnesium (GRIN2A, GRIN2B); [2] the calcium channel, voltage-dependent, gamma subunit 2, regulating the trafficking, and gating properties of AMPA-selective glutamate receptors (AMPARs), (CACNG2); [3] the discs, large (Drosophila) homolog-associated protein 1; Part of the postsynaptic scaffold in neuronal cells (DLGAP1); and [4] the calcium/calmodulin-dependent protein kinase II gamma, which may function in dendritic spines and synapse formation, as well as neuronal plasticity (CAMK2G). These associations place ADNP in a network regulating key neuronal processes (Fig. 5, Supplemental Table S1). Notably, analysis of the mouse database added calmodulin 1, mediating the control of a large number of enzymes, ion channels, aquaporins and other proteins by Ca(2 + ) and the calcium/calmodulin-dependent protein kinase II alpha. CaM-kinase II (CAMK2) is a prominent kinase in the central nervous system that may function in long-term potentiation and neurotransmitter release. As a member of the NMDAR signaling complex in excitatory synapses, it may regulate NMDAR-dependent potentiation of the AMPAR and synaptic plasticity (https://string-db.org/cgi/network.pl?taskId = gIL9cWwwcevm).

## Discussion

We show here, for the first time, substantial findings implying that the change of the preservative component in the NAP (davunetide) formulation (NAP-CB vs. NAP-benzaokonium—DD) resulted in: [1] brain vs. body concentration and [2] 4-fold increase in brain bioavailability, compared with the routinely used placebo, benzalkonium chloride. As the chlorobutanol preservative is used in men for > 125 years in other formulations for versatile uses, including nasal administration, the transition to clinical application is immediate. NAP-CB treatment resulted in complete amelioration of *Adnp* haploinsufficient cognitive deficits measured by the object recognition and the social memory tests.

Mouse sex specific variances included differences in hanging wire performance, as well as in the response to olfactory cues. Interestingly, we have previously shown increased resilience in females vs. males in the hanging wire performance in an ALS mouse model^5^, which was further corroborated by a later comparative study^59^. Regarding sex differences in odor discrimination observed here, a very recent study suggests that this depends on gonadal steroids^60^, and here we add potential regulation by NAP, in a sex-dependent manner. Furthermore, our results (Fig. 2) were corroborated in a recent study using the DD vehicle^40^. The significant reduction in the VGLUT1 mRNA transcripts in the male, but not in the female hippocampus suggests a more severe hippocampal phenotype in males due to *Adnp* deficiency. Similar findings were previously reported at the level of the presynaptic dendritic spines^40^. Importantly, these reductions were completely ameliorated by NAP treatment.

Our results went beyond behavioral outcomes and protein expression to measured changes in DTI, revealing significant NAP protection of brain matter, which could be further extended to measures of connectivity/gray matter intactness. We discovered here that deficits occurring as a consequence of *Adnp* deficiency in VGLUT1 gene expression were ameliorated by NAP treatment. In this respect, VGLUT1 has been previously linked to synapse density and by longitudinal imaging to subhippocampal dynamics associated with histopathologic events in mice^55^. Together, these studies suggest a molecular mechanism for the DTI observed changes, with NAP/ADNP previously linked to dendritic spine formation in vitro^2^ and in vivo^40^, as well as protection against excitotoxicity^1^.

The ADNP association with VGLUT1 and PSD95 also revealed a link to calcium regulation. Importantly, ADNP/NAP was previously shown to regulate calcium channel expression^8^, and exhibited association with major neuronal networks^40^.

The sex-specific impact on the glutamatergic system in the male may be linked to increased sensitivity of the male phenotype to *Adnp* deficiency, which was also observed in animals treated with the DD vehicle^40^. Interestingly, VGLUT1 is linked to epilepsy^61^, and some of the ADNP children indeed suffer from epilepsy^62^. Furthermore, NAP treatment was shown to provide neuroprotection in association with induction of epilepsy in a rodent model^19^.

From a translational neuroscience point of view, DTI can be implemented in humans as a measure of gray matter intactness^63^, as well as a prognostic tool for drug activity. Clinical trials with NAP (davunetide, CP201) in schizophrenia and Alzheimer’s disease (amnestic mild cognitive impairments) have shown protection of activities of daily living^23^, brain metabolism^24^, and increased cognitive function^64^, respectively. Previous studies described the diagnostic value of blood ADNP in Alzheimer’s disease^34^ and schizophrenia^4^, including risk ADNP SNPs in bipolar disorder with comorbid eating disorder^35^. Further studies showed the association of ADNP^65^ with schizophrenia mutated genes^37^ and ADNP regulation of the major risk gene for Alzheimer’s disease in a sex-dependent manner^30^ (detailed in the introduction). Together with our current findings, these investigations pave the path to patient stratification toward personalized medicine, with NAP-CB (also known as davunetide or CP201) as a lead candidate. Furthermore, while our recent paper suggested NAP-DD for the treatment of the *ADNP* syndrome^40^, the current paper suggests NAP-CB as a pipeline product.

The application of NAP-CB goes beyond brain protection. For example, NAP provided protection against inflammation in a model of Ileitis^66^ and the novel formulation may result in better efficacy in inflammatory bowel disease, as well as other inflammatory diseases for NAP and pipeline products^8^, affecting brain health and meeting unmet, highly prevalent, devastating and costly societal needs.

## Supplementary information

Supplemental Material
