# Supplementary material for: The autism/neuroprotection-linked ADNP/NAP regulate the excitatory glutamatergic synapse
Source: Transl Psychiatry. 2019 Jan 15;9:2. doi: 10.1038/s41398-018-0357-6 (PMC6341082; doi:10.1038/s41398-018-0357-6)
Supplement: Supplementary file 1 — Supplemental Material [file 41398_2018_357_MOESM1_ESM.docx]

**Supplementary Materials:**

**The Autism/Neuroprotection-Linked ADNP/NAP Regulate the Excitatory Glutamatergic Synapse**

Shlomo Sragovich^1*^, Anna Malishkevich^1*^, Yael Piontkewitz^2^, Eliezer Giladi^1^, Olga Touloumi^3^, Roza Lagoudaki^3^, Nikolaos Grigoiadis^3^ and Illana Gozes^1**^

^*^Contributed equally

^1^Lily and Avraham Gildor Chair for the Investigation of Growth Factors; Elton Laboratory for Neuroendocrinology; Department of Human Molecular Genetics and Biochemistry, Sackler Faculty of Medicine, Sagol School of Neuroscience and Adams Super Center for Brain Studies, Tel Aviv University, Tel Aviv, Israel. ^2^The Alfredo Federico Strauss Center for Computational Neuroimaging, Tel Aviv University, Tel Aviv, Israel. ^3^Department of Neurology, Laboratory of Experimental Neurology, AHEPA University Hospital, Aristotle University of Thessaloniki, Thessaloniki, Greece.

^**^**Corresponding author:**

Illana Gozes, Ph.D.; Professor of Clinical Biochemistry

The Lily and Avraham Gildor Chair for the Investigation of Growth Factors

Head, the Dr. Diana and Zelman Elton (Elbaum) Laboratory for Molecular Neuroendocrinology

Sackler Faculty of Medicine, Tel Aviv University

Tel Aviv 69978, Israel, Phone : 972-3-640-7240, Fax : 972-3-640-8541

E-mail : [igozes@tauex.tau.ac.il](mailto:igozes@tauex.tau.ac.il)

**Supplemental Figures:**

**
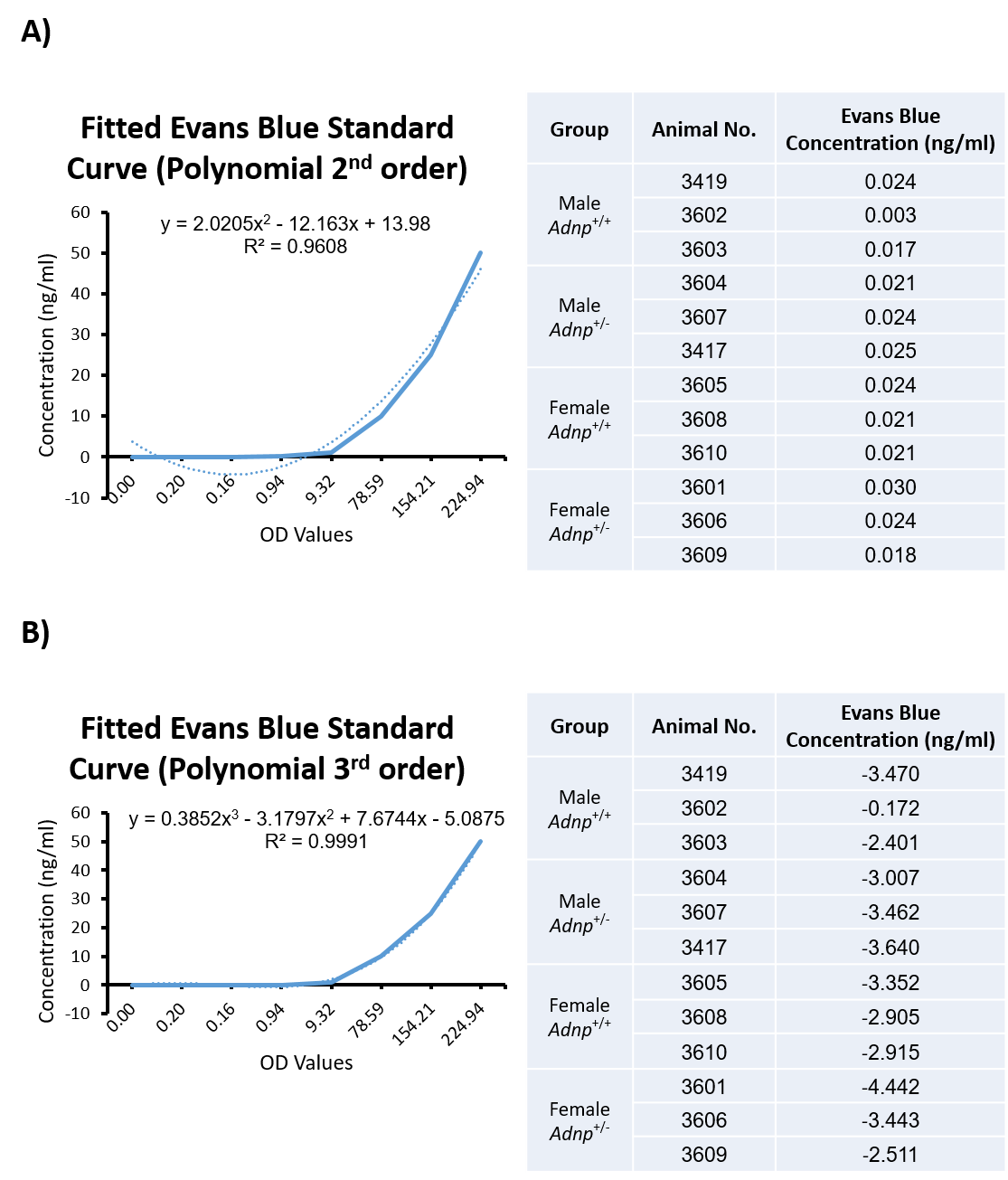
**

**Supplemental Figure S1**. **Evans blue dye does not penetrate the blood brain barrier (BBB) in the presence of CB.**

Fitted Evans blue polynomial standard curves with 2^nd^ order **(A)** and 3^rd^ order **(B)** are presented. The tables on the right side depict the Evans blue dye concentration (ng/ml) obtained for each animal in the experimental groups (n=3 per group). All the animals were administered with CB as detailed in the main text. The extrapolated values in both tables (either close to zero or negative) indicate that the Evans blue dye does not penetrate the BBB. No significant genotype or sex differences were found among the experimental groups.

**
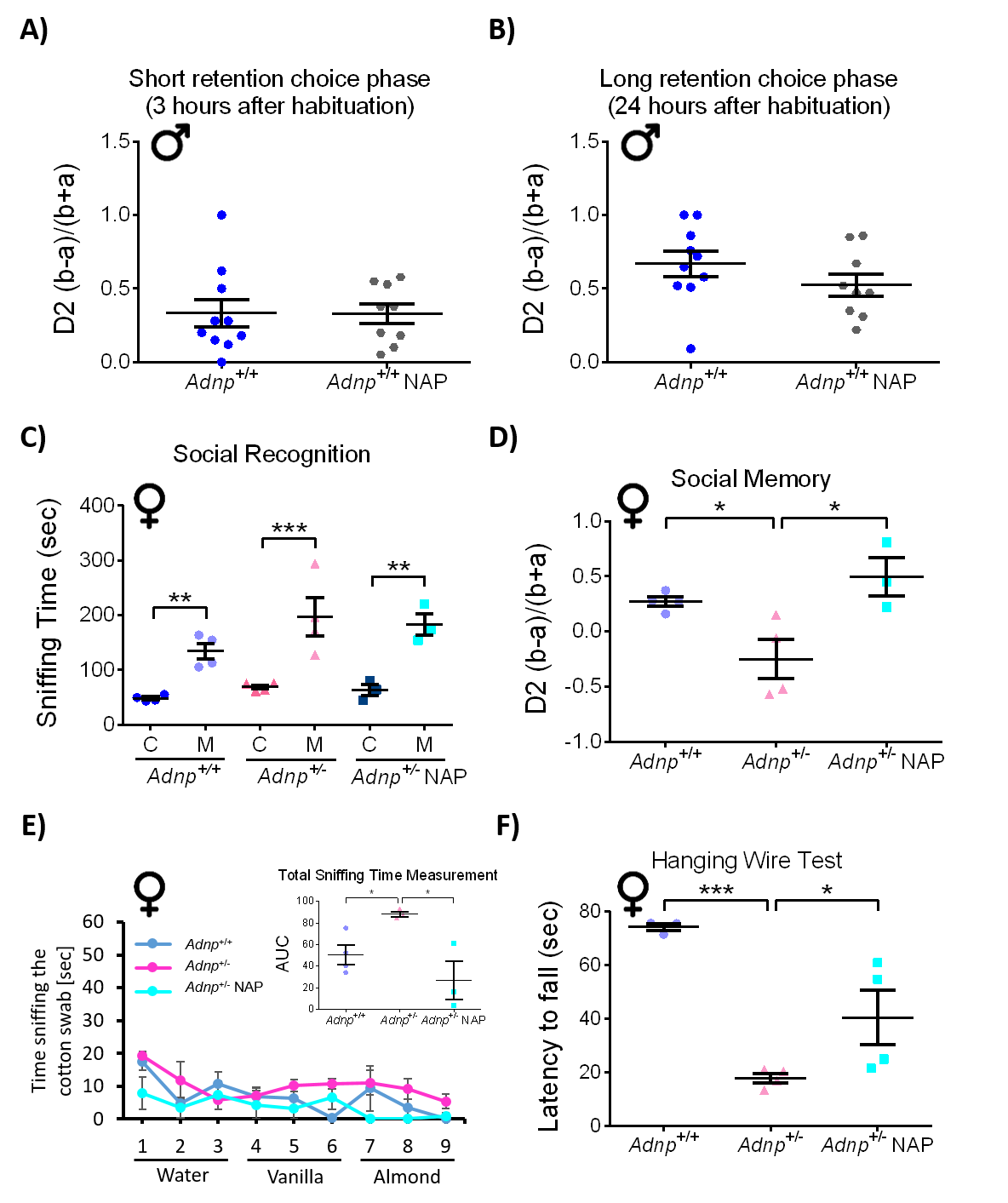
**

**Supplemental Figure S2. NAP treatment does not affect relative discrimination between novel and familiar objects in *Adnp*^+/+^ male mice, and protects social memory and motor function in *Adnp*^+/-^ females.**

For females, animal performance in the behavioral tests is shown (n=3-4 per experimental group). Data are expressed as mean (±SEM) total time (sec) spent exploring all objects/mice designated by relative discrimination index (D2, a and b - exploration of familiar and novel objects/mice, respectively).

**(A-B)** For both short and long retention choice phases in males, no significant differences were observed between NAP- and CB-treated *Adnp*^+/+^ mice.

**For females: (C)** In the social recognition test, main effect for sniffed item was found (F(1,8)=55.240, p<0.001), with significant differences between sniffing time of the cup (C) and mouse (M) in *Adnp*^+/+^, *Adnp*^+/-^, and NAP-treated *Adnp*^+/-^ mice (**p<0.01, ***p<0.001 vs. cup). Two-way repeated measures ANOVA with group as a fixed factor and sniffed item (e.g. mouse vs. cup) as repeated factor and Tukey post hoc test was performed. **(D)** *Adnp*^+/-^ female mice spent less time in exploring the novel mouse, as compared with *Adnp*^+/+^ mice. Treatment with NAP improved social memory for the *Adnp*^+/-^ mice. Unpaired student's t-test analyses revealed significant differences between vehicle-treated *Adnp*^+/+^ and *Adnp*^+/-^ mice, and between NAP- and vehicle-treated *Adnp*^+/-^ mice (***p<0.001). All reported p-values were also significant after multiple comparisons correction at FDR of 10%. **(E)** In females, no significant differences were found between experimental groups in odor discrimination ability, when comparing novel vs. familiar odor. For each experimental group, general olfaction ability was measured by calculating area under the curve (AUC) - inset graph. Unpaired student's t-test analyses revealed significant differences between vehicle-treated *Adnp*^+/+^ and *Adnp*^+/-^ mice, and between NAP- and vehicle-treated *Adnp*^+/-^ mice (*p<0.05), with increased total sniffing time in *Adnp*^+/-^ females. **(F)** In the Hanging Wire Test, female *Adnp*^+/-^ mice display significant decreased latency to fall, with NAP treatment ameliorating this behavior. Unpaired student's t-test analyses revealed significant differences between vehicle-treated *Adnp*^+/+^ and *Adnp*^+/-^ mice, and between NAP- and vehicle-treated *Adnp*^+/-^ mice (***p<0.001, *p<0.05). All reported p-values were also significant after multiple comparisons correction at FDR of 10%.


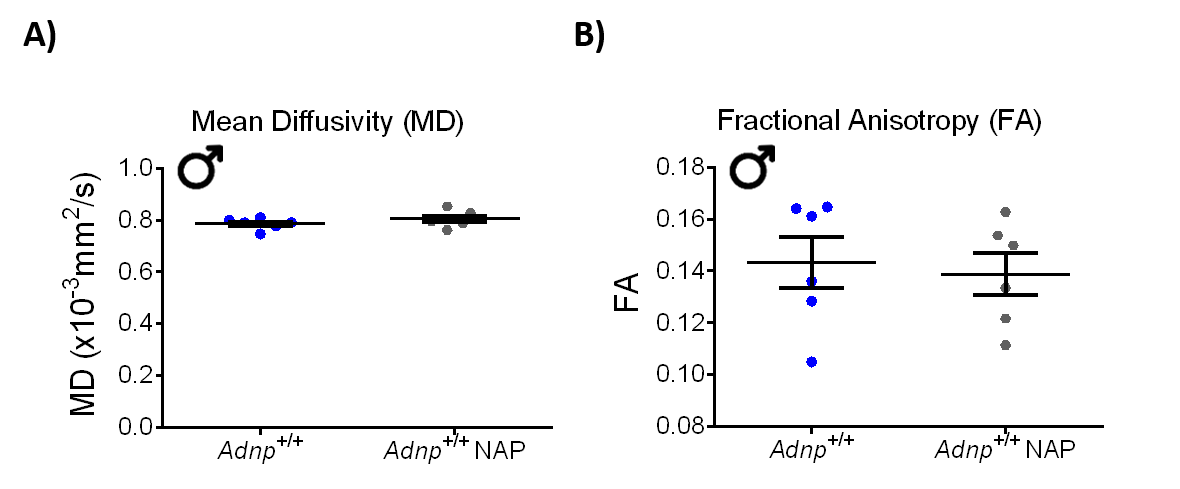


**Supplemental Figure S3. In males, NAP does not affect hippocampal mean diffusivity (MD) and fractional anisotropy (FA) in the *Adnp*^+/+^ mice.**

Two-way ANOVA with Tukey post hoc test was performed (n=4-6 per experimental group). **(A)** In a hippocampal area located -1.84 mm from Bregma, main genotype (F(1,16)=8.775, p=0.009) and interaction (F(1,16)=4.956, p=0.041) effects were found, with no significant differences in MD between NAP- and CB-treated *Adnp*^+/+^ mice. **(B)** In a hippocampal area located -2.34 mm from Bregma, main treatment (F(1,16)=12.782, p=0.003) and interaction (F(1,16)=9.986, p=0.006) effects were found, with no significant differences in FA between NAP- and CB-treated *Adnp*^+/+^ mice.


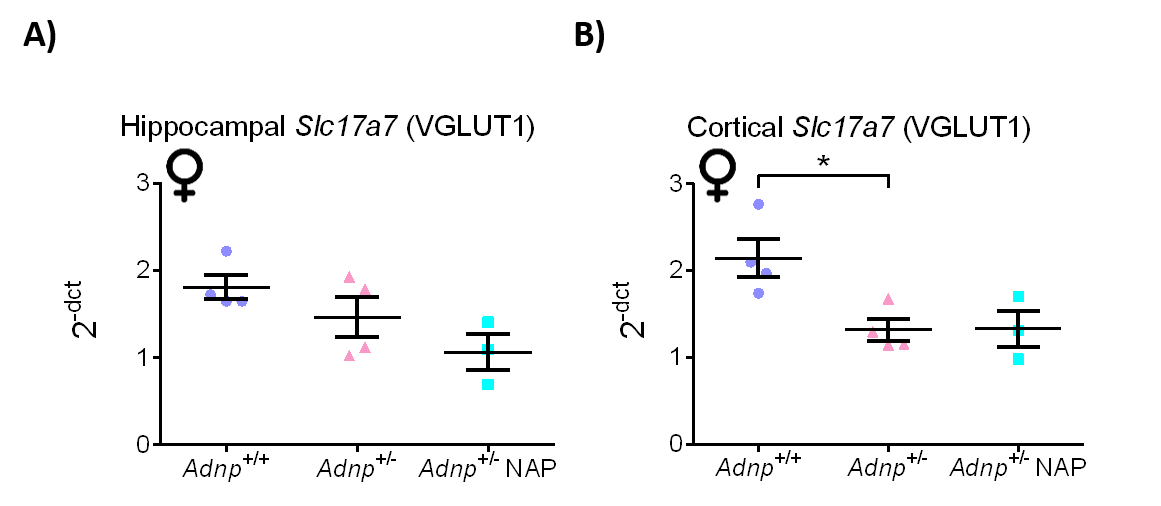


**Supplemental Figure S4. In females, *Adnp* deficiency results in cortical *Slc17a7* (VGLUT1) gene expression alterations.**

Results are presented as 2^-ΔCT^, normalized to *Hprt* (n=3-4 per experimental group). **(A)** In the female hippocampus, no significant differences were found in VGLUT1 gene expression among the experimental groups. **(B)** In the female cerebral cortex, unpaired student's t-test analyses revealed a significant difference between CB-treated *Adnp*^+/+^ and *Adnp*^+/-^ mice (*p<0.05), with no significant effect for NAP treatment. All reported p-values were also significant after multiple comparisons correction at FDR of 10%.

**Supplemental Materials and Methods:**

Object recognition: The test included two consecutive days of habituation (5 min per day) and the experimental day which consisted of the three phases. In phase 1 (habituation), the open field apparatus (50 × 50 cm) contained two identical objects (plastic or metal, 4 × 5 cm^2^) and a mouse was placed in the apparatus facing the wall and allowed to freely explore the objects (5 min). After 3 h in the home cage, the mouse was placed back into the apparatus for 3 min for phase 2 (short retention choice phase), during which one of the familiar objects was replaced with a novel object. Approximately 24 h after the completion of phase 2, the mouse was placed into the apparatus for 3 min for phase 3 (long retention choice phase), during which one of the familiar objects was replaced with a novel object. The mouse was kept in its home cage between phases 2 and 3. The time spent sniffing/touching each object was measured. Mouse movement and exploratory behavior were tracked and recorded using the EthoVision XT video tracking system and software (Noldus Inc. Leesburg, VA). Data were analyzed using the discrimination capacity formula: D2 = (b-a)/(a+b), where ‘a’ designated the time of exploration of the familiar object and 'b' designated the time of exploration of the novel object.

Social approach: A plexiglas box was divided into three adjacent chambers, each 20 cm (length) × 40.5 cm (width) × 22 cm (height), separated by two removable doors. Steel wire pencil cups (10.16 cm (diameter), 10.8 cm (height)), www.kitchen-plus.com, were used as both containment for the target mice and as inanimate objects (weights prevented the mice from overturning the cups). Experiments were conducted in a dimly lit area during the light phase of the mouse. Target mice (males for males and females for females) were placed inside the wire cup in one of the side chambers for three 10-min sessions on the day before the test for habituation. The next day, each subject mouse was tested in an experiment with three phases, each 10-min long (measured with a simple timer): I and II, the habituation phases (ensuring no bias), and III, the experimental phase. No significant differences were noted between time periods spent in the different chambers in the habituation phase. In phase III, an empty wire cup (novel object) was placed in the center of the right or left chamber and the cup containing the target mouse was placed in the center of the other chamber. Location of the empty wire cup (novel object) and the novel mice were counterbalanced to avoid confounding side preference. The doors were then removed and a 10-min timer was initiated. The three-chamber apparatus was cleaned between mice. The social approach task was also used as habituation for the social memory task, 3 h after the first phase (3-min exposure), the mouse was placed back into the apparatus for another 3 min (second phase), during which one cup contained the familiar mouse and the other contained a novel mouse. The positions of the familiar and novel mouse during phases 1 and 2 were counterbalanced within and between groups to exclude the possibility of positional effects but were kept the same for a given animal. Mouse movement and exploratory behavior were tracked and recorded using the EthoVision XT video tracking system and software (Noldus Inc. Leesburg, VA). The discrimination capacity (social memory) was analyzed using the formula: D2 = (b-a)/(b+a), where ‘a’ designated the time of exploration of the familiar mouse and 'b' designated the time of exploration of the novel mouse.
